# Supplementary material for: Controlled and orthogonal partitioning of large particles into biomolecular condensates
Source: Nat Commun. 2025 Apr 14;16:3521. doi: 10.1038/s41467-025-58900-5 (PMC11997106; doi:10.1038/s41467-025-58900-5)

Supporting Information for

Controlled and orthogonal partitioning of large particles into biomolecular condensates

Fleurie M. Kelley[1], Anas Ani[1,2], Emily G. Pinlac[1], Bridget Linders[1], Bruna Favetta[3], Mayur Barai[1], Yuchen Ma[2], Arjun Singh[1], Gregory L. Dignon[1], Yuwei Gu[2], Benjamin S. Schuster[1]

1. Department of Chemical and Biochemical Engineering, Rutgers, the State University of New Jersey, Piscataway, NJ, 08854, USA;
2. Department of Chemistry and Chemical Biology, Rutgers, the State University of New Jersey, Piscataway, NJ, 08854, USA;
3. Department of Biomedical Engineering, Rutgers, the State University of New Jersey, Piscataway, NJ, 08854, USA;

Corresponding authors:
Benjamin S. Schuster. Email: benjamin.schuster@rutgers.edu
Yuwei Gu. Email: yuwei.gu@rutgers.edu
Gregory L. Dignon. Email: gregory.dignon@rutgers.edu

This PDF file includes:

Supplementary Figures 1 to 17
Supplementary Tables 1 to 8
Supplementary Notes
Supplementary References

Supplementary Figures

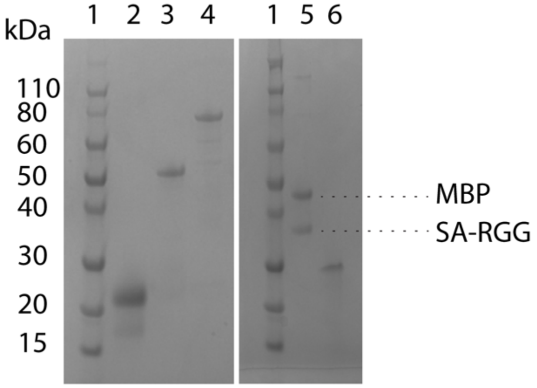

1. Ladder
2. RGG
3. N protein
4. MBP-SA-RGG, heated to 90°C
5. MBP-SA-RGG + TEV, heated to 90°C
6. $(GRGNSPYS)_{25}$

Figure 1. SDS-PAGE of purified proteins prepared for this study. MBP-SA-RGG + TEV refers to MBP-SA-RGG that had been treated overnight by TEV protease to cleave off the MBP and liberate SA-RGG. MBP-SA-RGG and MBP-SA-RGG + TEV samples were heated at 90°C prior to running the gel to monomerize streptavidin (SA), which tetramerizes at room temperature. All other samples were prepared for running the gel by heating to 70°C.

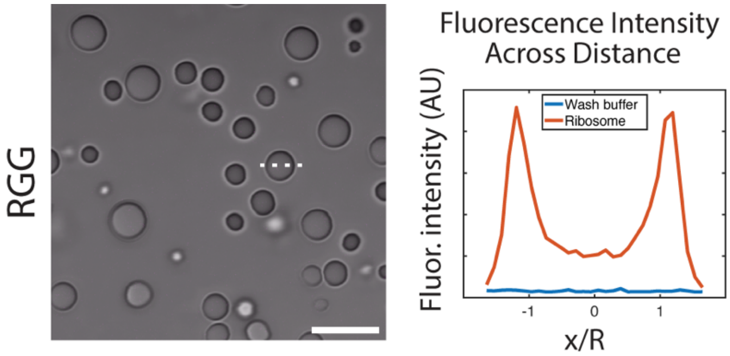

Figure 2. Partitioning of DL650-labeled ribosomes into RGG condensates, compared to partitioning of wash buffer following the ribosome labeling reaction. (Left) Partitioning of wash buffer into RGG. The partitioning of DL650-labled ribosomes into RGG is shown in Fig. 1B. (Right) Fluorescence intensity (not normalized) across distance, comparing DL650-ribosomes (red) and wash buffer (blue). The purpose of this comparison is to show that unbound dye was thoroughly removed from the DL650-labeled ribosomes, so the fluorescent signal shown in Fig. 1B is from the labeled ribosomes and not unbound dye.

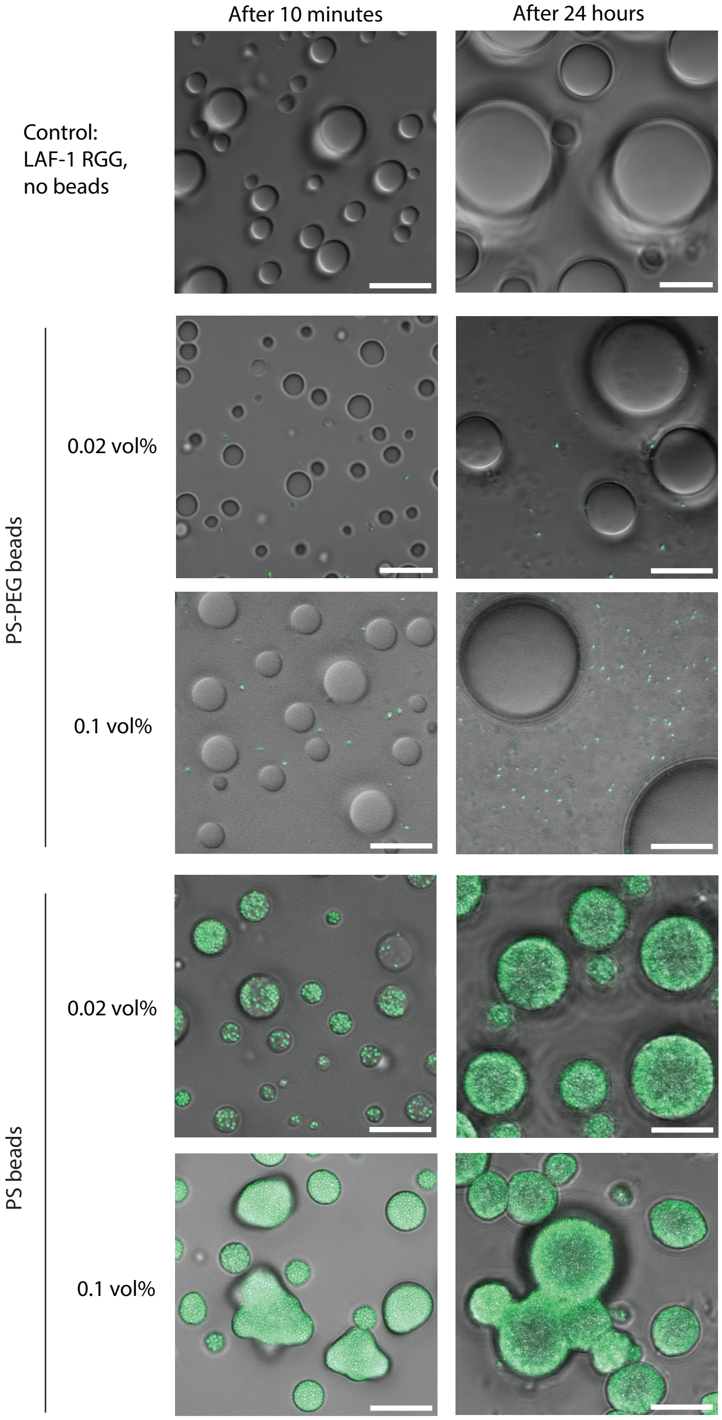

Figure 3. Effect of bead concentration and surface chemistry on condensate morphology and stability. LAF-1 RGG condensates were mixed with different concentrations of PS and PS-PEG beads (500 nm) and imaged after 10 minutes and after 24 hours. RGG condensates appear to aggregate when exposed to high (0.1 vol%) concentration of the sticky PS beads, whereas high concentration of PS-PEG beads do not appear to affect condensate morphology. Lower bead concentrations (0.02 vol%) did not appear to alter condensate morphology or stability.

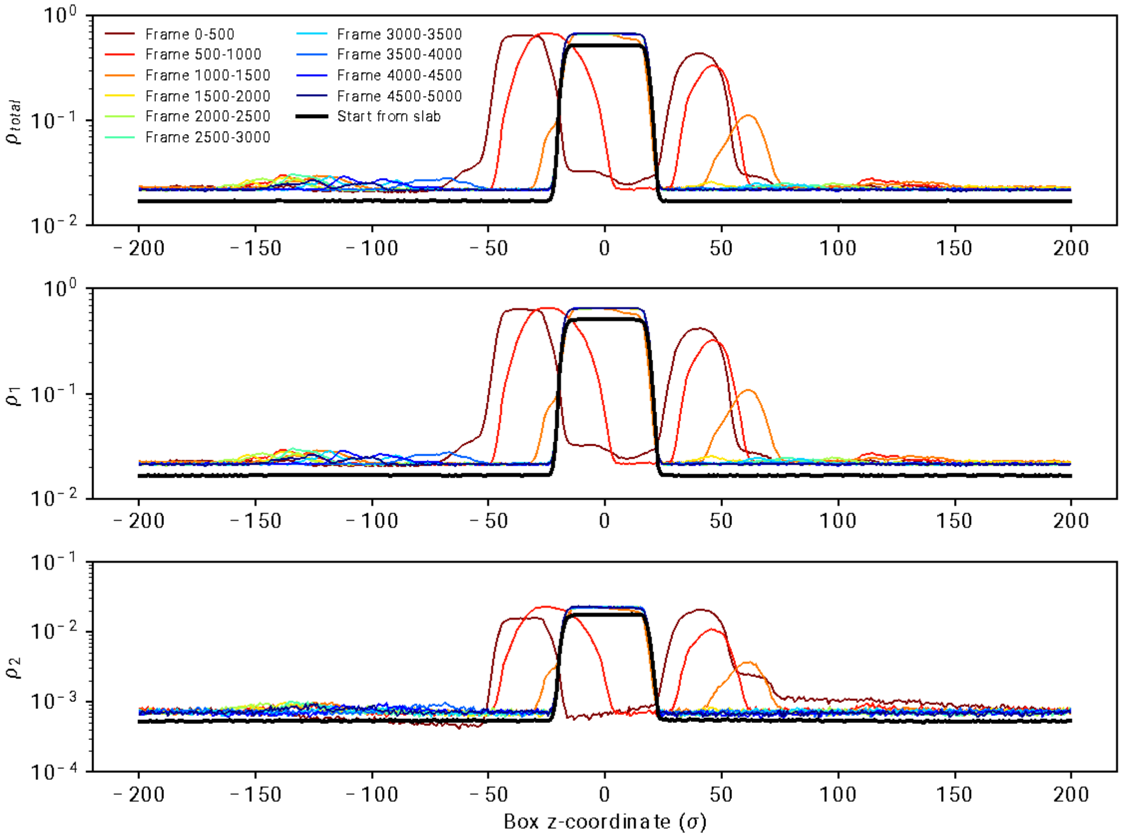

Figure 4. Effect of different starting conformations on convergence of simulation. The three plots show all molecules (top), only protein (1) molecules (middle), and only bead (2) molecules (bottom). When simulations were initiated with beads dispersed through both sides of the box, rather than clustered toward the center of the box, they rapidly coalesce into multiple slabs, seen up to Frame ~1500 (18 million MD timesteps). After this point, the two slabs combine and form a single slab and show a similar density profile to the system that was initialized in a slab-like configuration. However, a second "condensate" is still present throughout the course of the simulation on the left side. This seems to have a small impact on the average concentration in the aqueous and condensed phases, increasing both the high and low equilibrium concentrations, ρ_{high} and ρ_{low}, by a small amount. We note that since both concentrations are increased, the impact on the partition coefficient is relatively small. From this, we conclude that starting from a dispersed configuration will eventually relax to the same equilibrium state as a system that is initialized as a slab-like configuration. By initializing as a slab-like configuration, we reduce the amount of simulation time required, while yielding results consistent with what would be observed if the simulation were initiated from a different starting point. These results are also consistent with tests done in previous work to show that starting configuration does not have an impact on the equilibrium density profile of the slab simulation.[1,2]

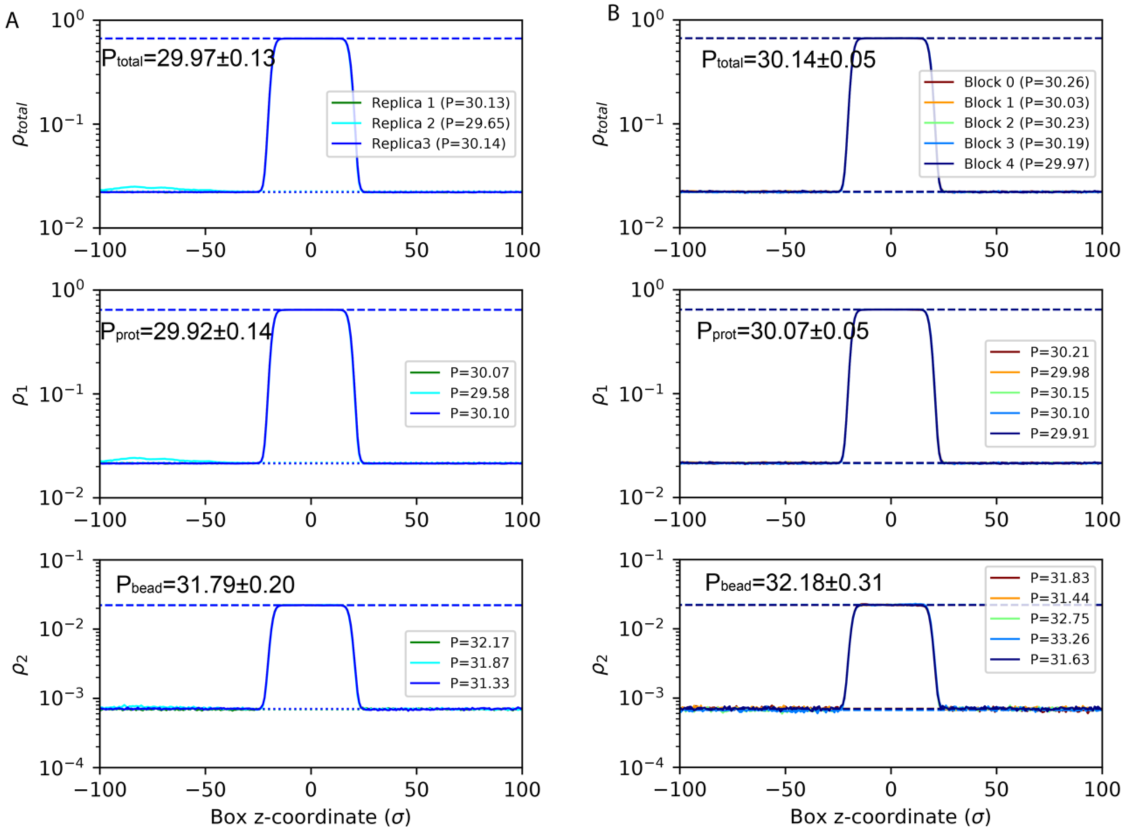

Figure 5. Multiple replicas vs. block averaging of simulation trajectories. A) Three separate replicas of the ε=1.2, σ=2 bead system were analyzed to show convergence of simulations. Partition coefficients of different components and their uncertainty can be quantified as SEM of the values from the three replicas. Partition coefficients (P) are calculated for the whole system and each individual component for all replicas, and averaged together in the inset with uncertainty reported as SEM. B) Splitting a single trajectory into 5 blocks allows for calculation of SEM of the partition coefficients from the single trajectory. The values of uncertainty from both methods are of a similar magnitude. Partition coefficients for each density profile and average and SEM values are calculated in the same way as in subplot A.

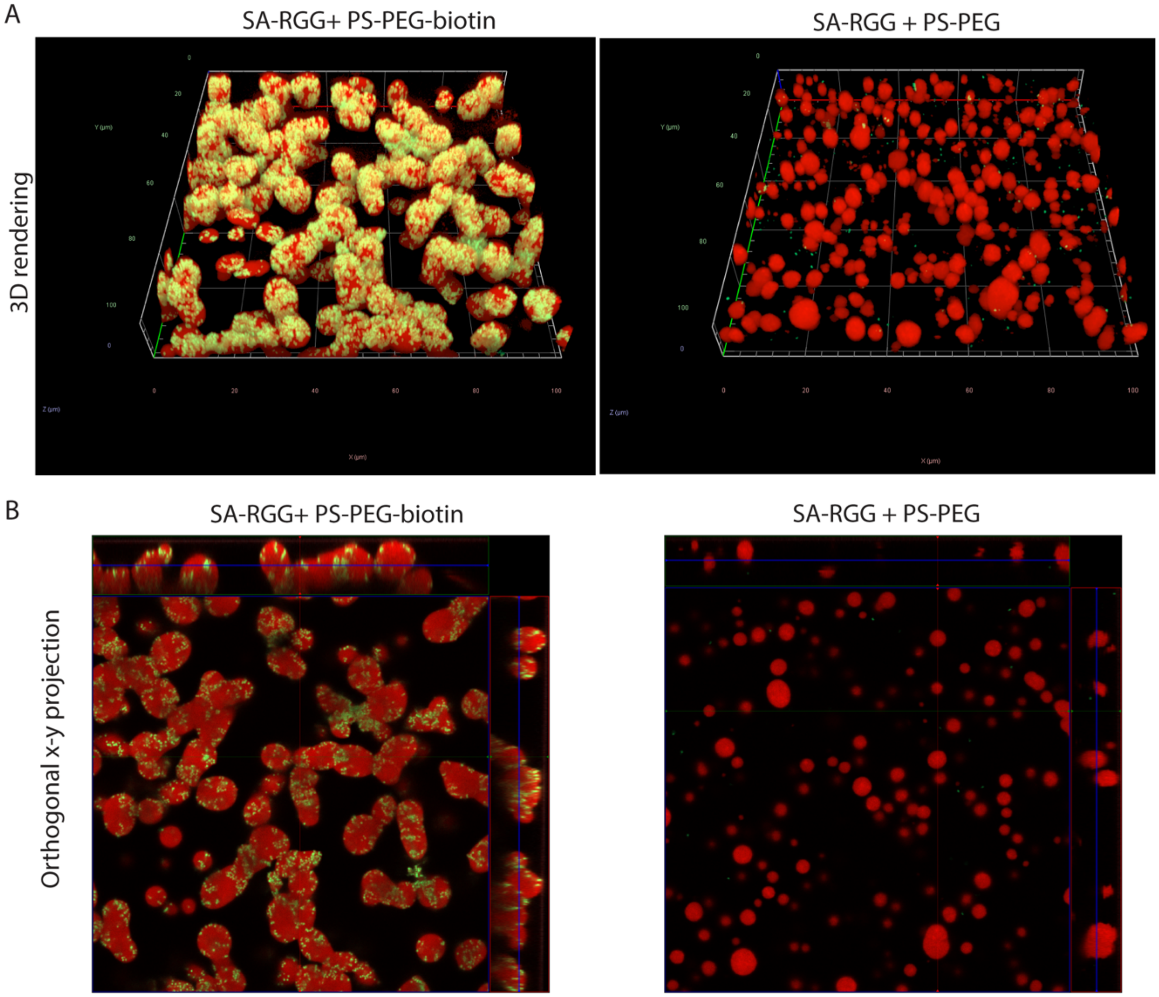

Figure 6. Confocal microscopy of SA-RGG + PS-PEG-biotin vs. SA-RGG + PS-PEG. Rhodamine (red) was added to visualize the SA-RGG condensates. Nanoparticles (500 nm diameter) are green. (A) 3D renderings from z-stacks of SA-RGG + PS-PEG-biotin (left) and SA-RGG + PS-PEG (right). (B) Orthogonal projections showing xy, xz, and yz planes.

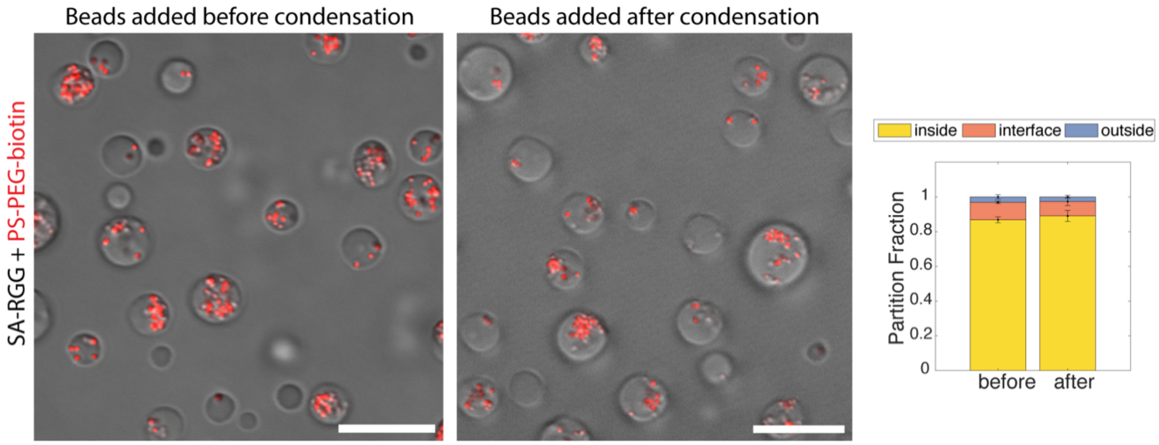

Figure 7. PS-PEG-biotin beads (500 nm) added to SA-RGG before vs. after phase separation of SA-RGG. The partition fraction of the beads into SA-RGG condensates is similar whether the beads were added before or after condensate formation. Error bars represent SEM with n = 8. For all figures, n equals number of images from at least 2 independent experiments, unless otherwise stated.

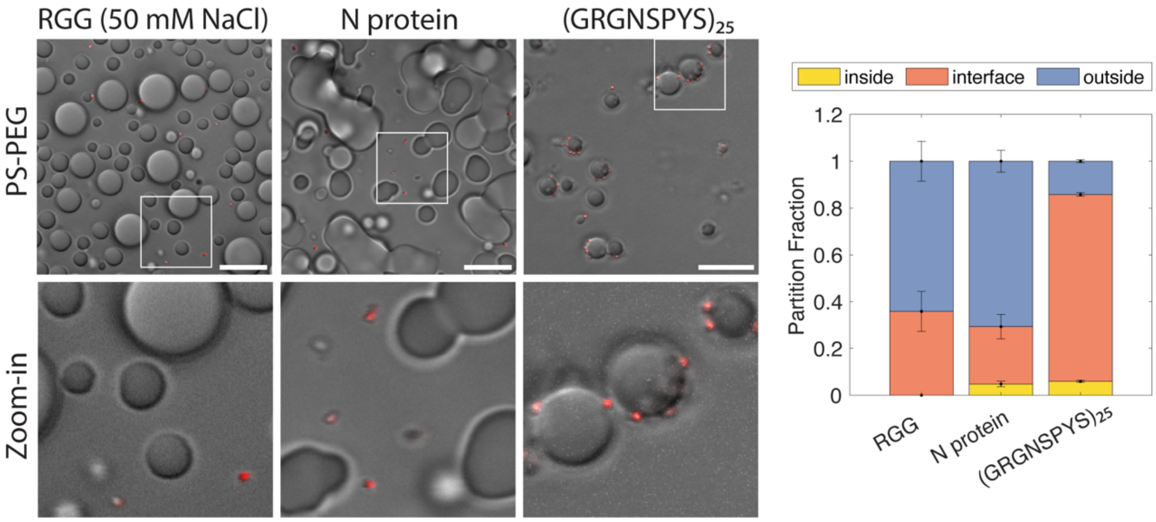

Figure 8. PS-PEG beads (500 nm) are excluded from RGG, N protein, and $(GRGNSPYS)_{25}$ condensates. Here, RGG was prepared in 50 mM NaCl buffer, and the other two proteins were prepared in 150 mM NaCl buffer. (Left) Microscopy images. (Right) Partition fractions were quantified. Error bars represent SEM with n = 10.

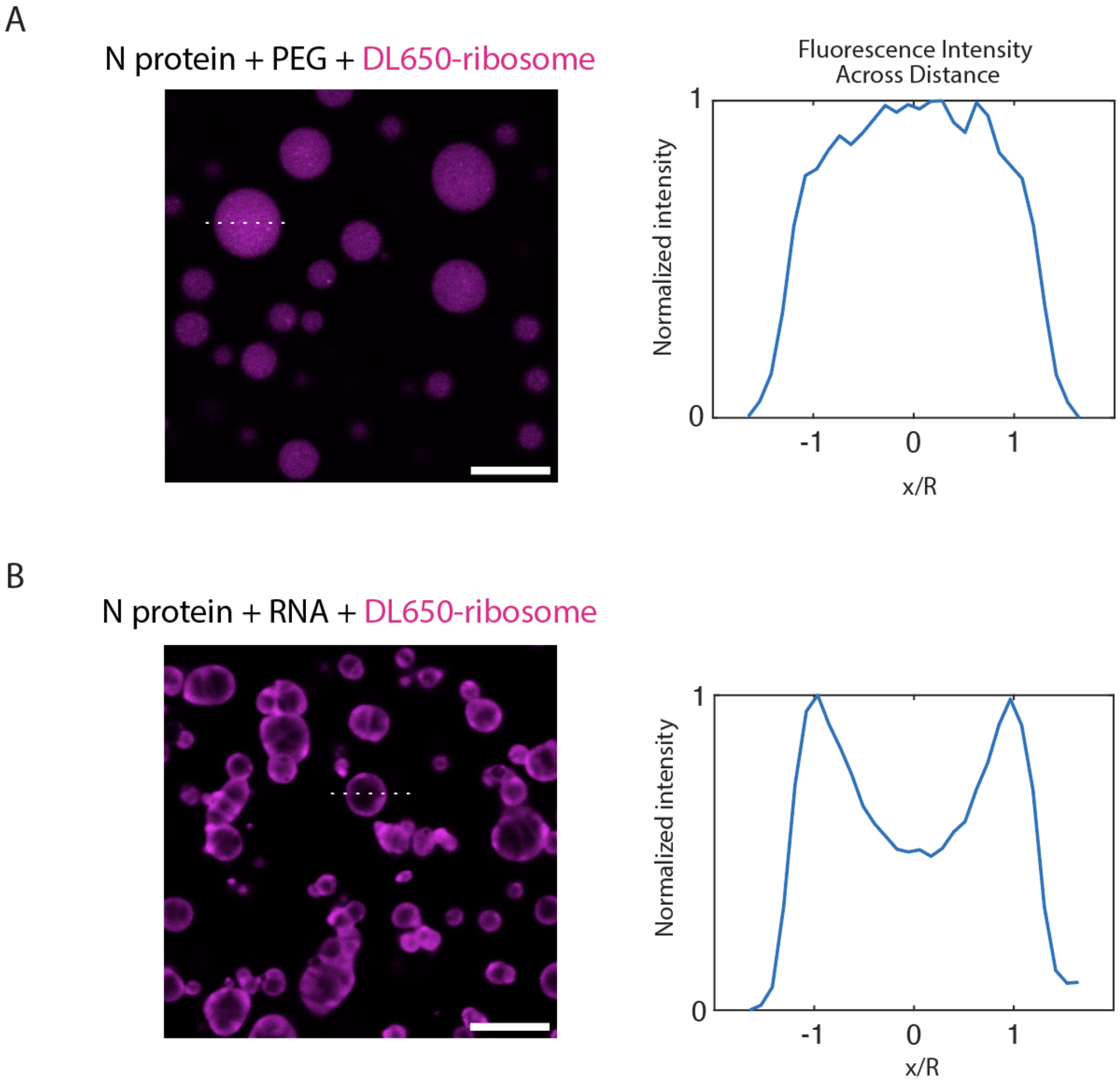

Figure 9. Partitioning of ribosomes into N protein condensates prepared with PEG vs. RNA. A) Ribosomes partition homogeneously into condensates prepared from N protein with 5% (w/v) 8 kDa PEG as a crowding agent. B) For condensates prepared with N protein + polyA RNA (the same conditions as in Fig. 1B; data copied here for the sake of comparison), ribosomes partition inhomogeneously and are concentrated towards the periphery of the condensates. Fluorescence intensities were quantified as line profiles across individual condensates, which were normalized and averaged, for each image.

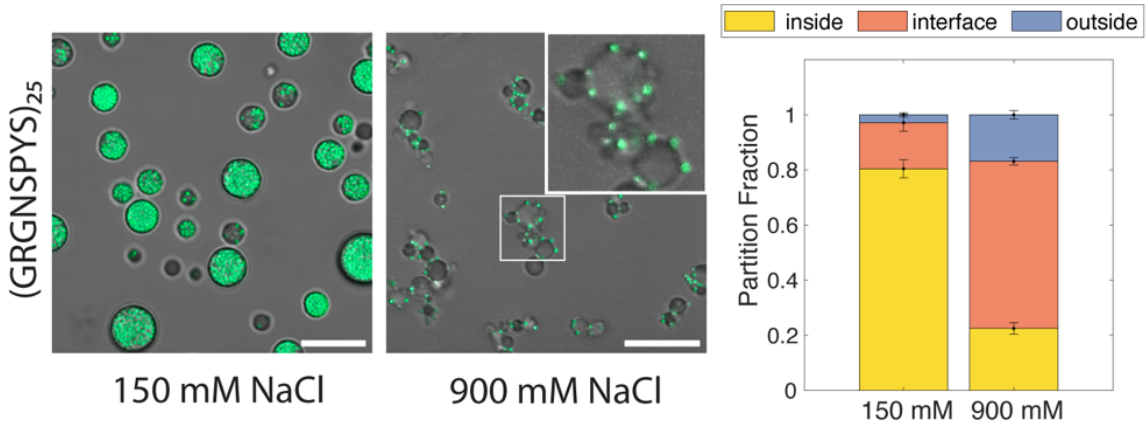

Figure 10. GRGNSPYS condensates with 500 nm PS-PEG-polyA20 beads (green) in 150 mM NaCl vs. 900 mM NaCl buffers (both with 20 mM Tris, pH 7.5)**.** (Left) Micrographs show beads are excluded from condensates at high salt concentration. Inset in 900 mM NaCl image shows zoom-in of boxed region. (Right) Partition fractions were quantified. Error bars represent SEM with n = 10.

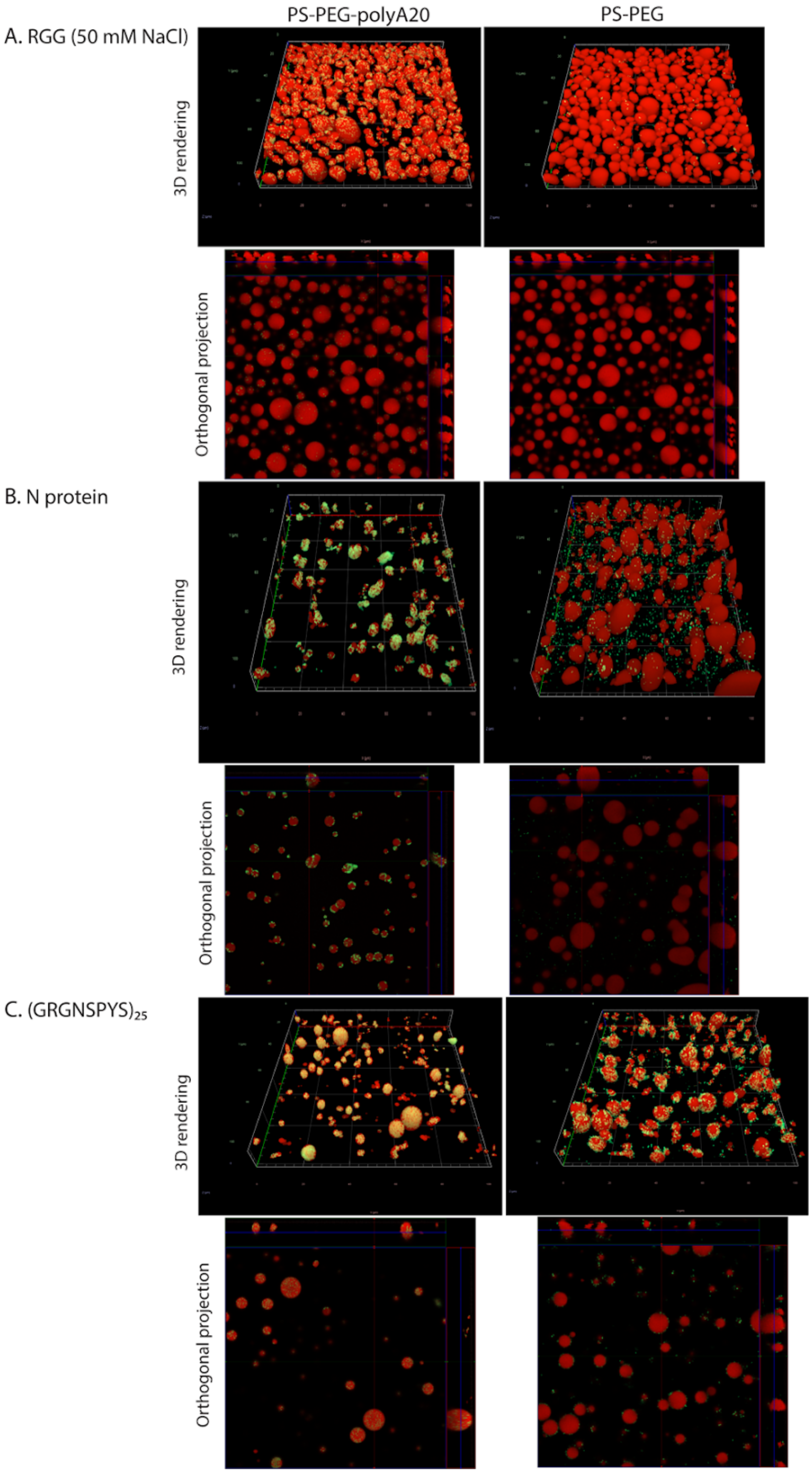

Figure 11. Confocal microscopy of condensates with PS-PEG-polyA20 vs. PS-PEG beads. Rhodamine (red) was added to visualize the condensates. Nanoparticles (500 nm diameter) are green. PS-PEG in this figure refers to PS-PEG-azide, where the free end of the PEG has an azide functional group, which was subsequently used for click chemistry reaction with DBCO-polyA20**.** (A) LAF-1 RGG at 50 mM NaCl. (B) N protein at 150 mM NaCl. (C) GRGNSPYS at 150 mM NaCl. For each protein, 3D renderings and orthogonal projections are shown.

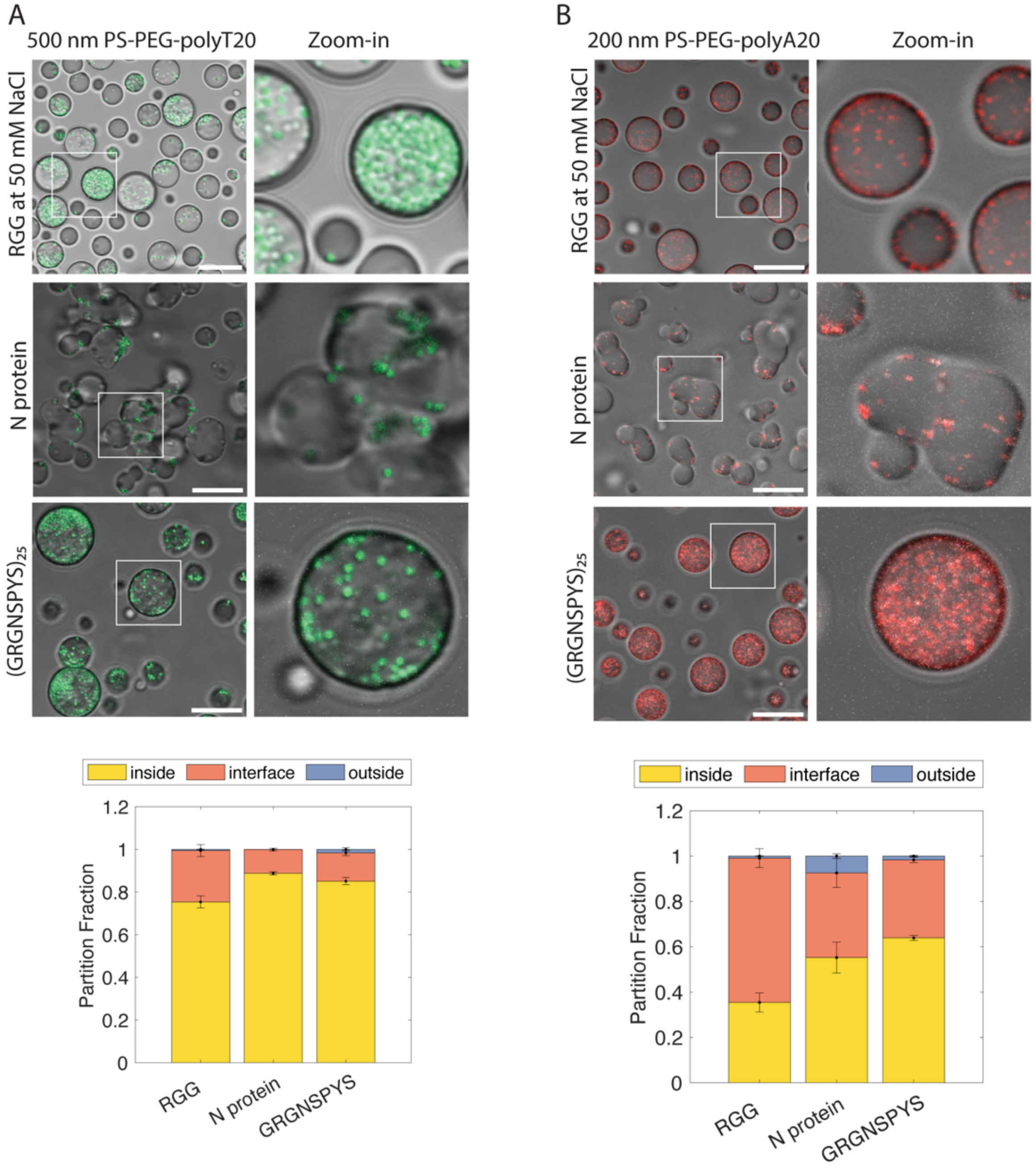

Figure 12. Additional oligonucleotide sequence (polyT20) and bead size (200 nm) tested for PS-PEG-oligo partitioning into condensates. (A) 500 nm PS-PEG-polyT20 beads partition into RGG (at 50 mM NaCl), N protein (at 150 mM NaCl), and GRGNSPYS (at 150 mM NaCl). (B) 200 nm PS-PEG-polyA20 beads also partition. Error bars represent SEM with n ≥ 5.

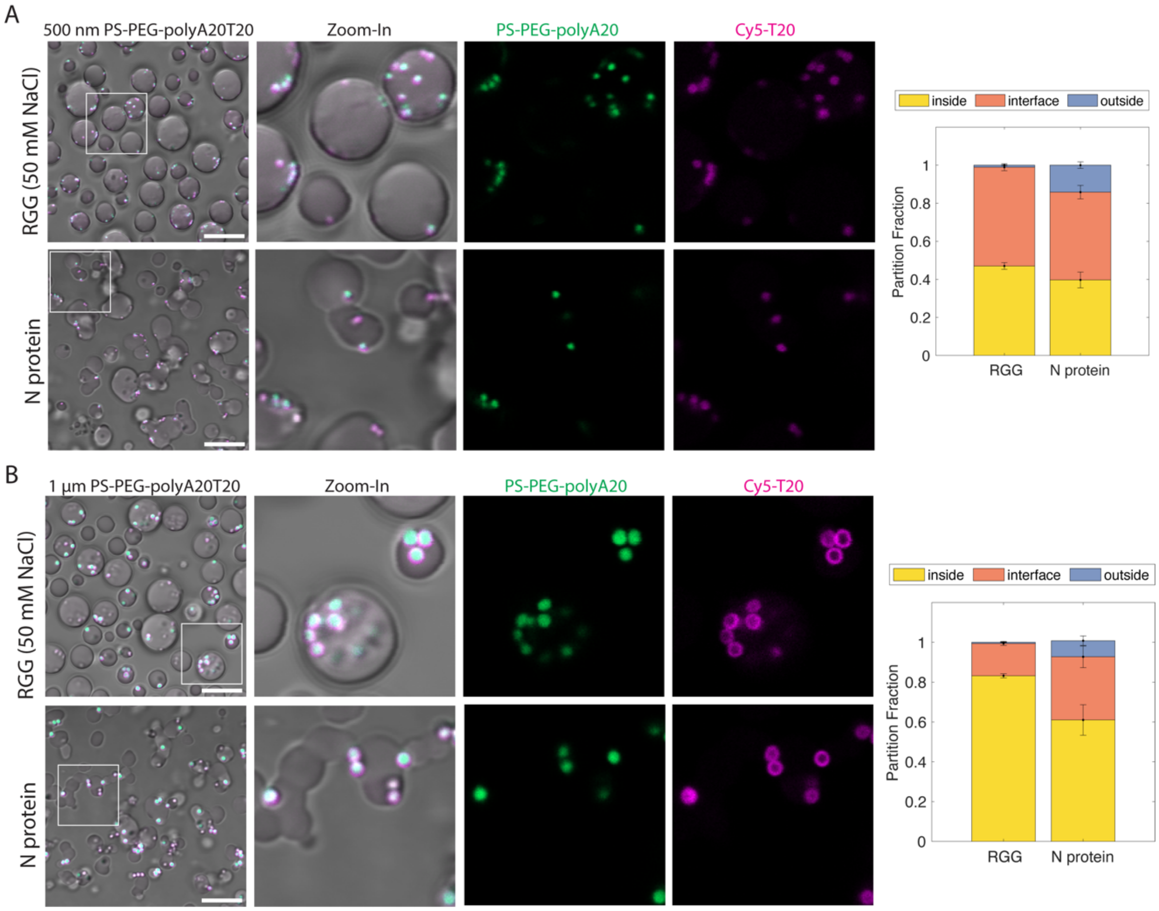

Figure 13. Beads conjugated with double-stranded oligonucleotides partition into condensates. RGG is in 50 mM NaCl buffer and N protein in 150 mM NaCl buffer. polyA20/T20 beads had been prepared as follows: polyA20 was conjugated to PS-PEG beads (green) by click chemistry, then Cy5-polyT20 was annealed to the PS-PEG-polyA20 beads. Colocalization of the Cy5 signal with the beads demonstrates successful annealing between polyT20 and PS-PEG-polyA20 to form PS-PEG-polyA20/T20. (A) Partitioning of 500 nm PS-PEG-polyA20/T20. (B) Partitioning of 1 µm PS-PEG-polyA20/T20. Scale bars represent 10 µm. Error bars represent SEM with n = 10.

PS-PEG-75%A20

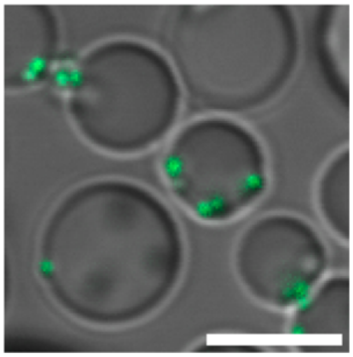

Figure 14. PS-PEG-75%polyA20 beads (500 nm) adhere to the interface of RGG condensates (in 50 mM NaCl buffer)**.** Partitioning is quantified in Fig. 4E.

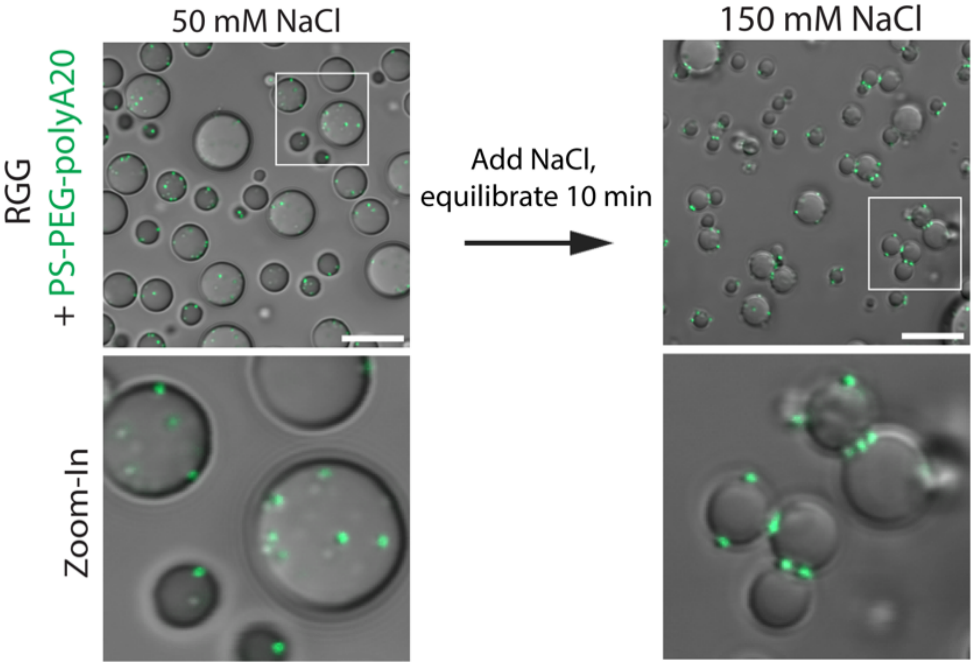

Figure 15. Reversing partitioning by increasing salt concentration. PS-PEG-polyA20 particles (green) partition into RGG condensates at 50 mM NaCl. When NaCl concentration is raised to 150 mM, previously partitioned beads become excluded from condensates. Scale bars, 10 μm. (Right) Partition fractions quantified. Error bars represent SEM with $n \geq 9$.

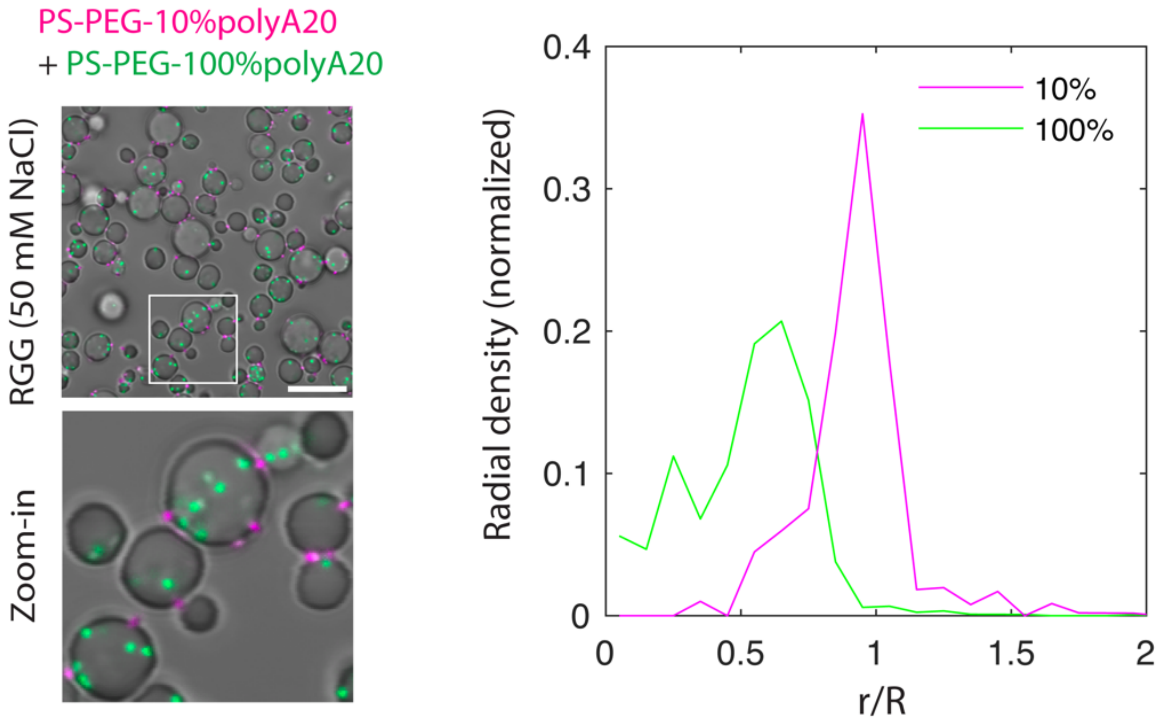

Figure 16. Selective targeting of condensate interior vs. interface based on oligo density on bead surface. Condensates are RGG at 50 mM NaCl**.** Bead diameter is 500 nm. Micrographs show PS-PEG-10%polyA20 beads (magenta) adsorb to the condensate interface and PS-PEG-100%polyA20 beads (green) are enriched inside condensates. (Right) Radial density profile shows bead concentration as a function of normalized distance from condensate center. (Scale bar, 10 μm.)

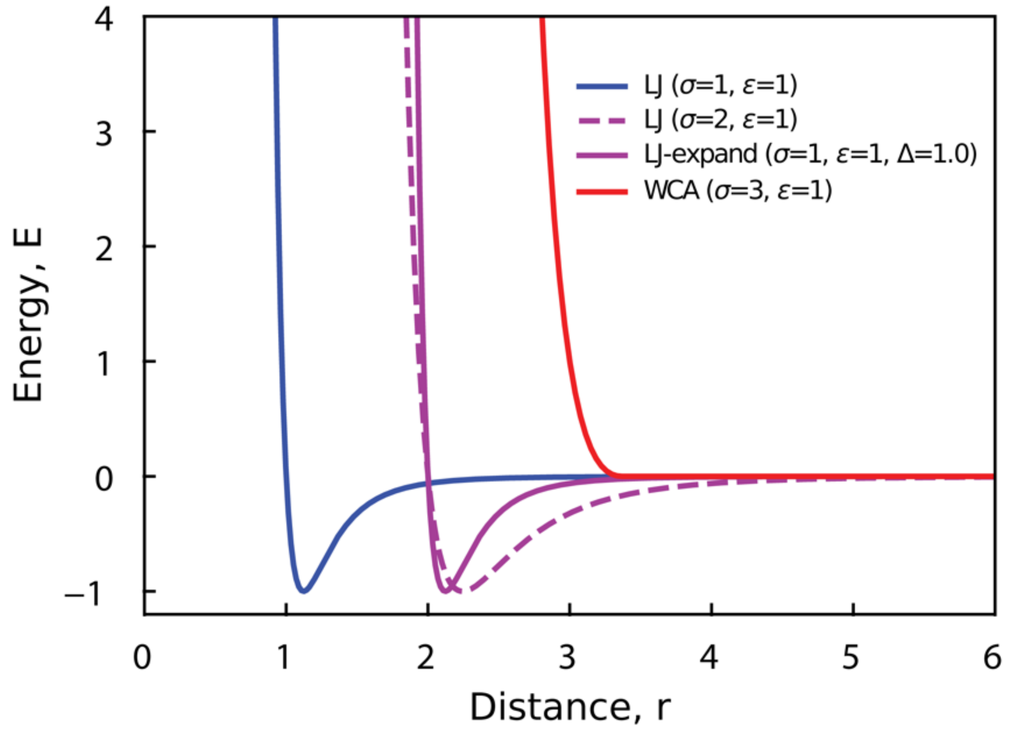

Figure 17. Energy functions for pairwise interactions between spherical particles used in molecular dynamics simulations. As an example, we are showing functional forms for the case where bead size is 3 (protein size is always 1, so for protein-bead interactions in this example, the average σ = 2 is used). The protein-protein interactions are a simple LJ functional form, while protein-bead interactions are handled by an LJ-expand functional form, which keeps the shape of the energy well the same as with protein-protein interactions, but shifts it outward to increase the effective size of the beads. For comparison, the LJ functional form for the same size is shown as a dashed line, illustrating the widening of the energy well. Finally, bead-bead interactions were treated repulsively using a Weeks-Chandler-Andersen (WCA) potential.

Protein	N_{aa}	<λ>	q	f+	f-	FCR	NCPR
RGG	176	0.571	3.0	0.136	0.119	0.255	0.017
N protein	433	0.586	23.0	0.139	0.085	0.224	0.053
GRGNSPYS	229	0.637	25.0	0.122	0.013	0.135	0.109
SA-RGG	311	0.587	3	0.106	0.096	0.202	0.010

Table 1. Sequence statistics for four proteins used in this study. N_{aa} is number of amino acids; <λ> is the average hydropathy of the sequence based on the Urry hydropathy scale (values range from 0 to 1, where 0 is the least hydrophobic); q is the net charge; f+ and f- are the fraction of positively and negatively charged residues in the sequence, respectively; FCR is the fraction of charged residues; and NCPR is the net charge per residue.

Measurements in water

Particle	Size (nm)	Standard Deviation (SD) (nm)	Polydispersity index (PDI)
200 nm PS	220	3.7	0.027
200 nm PS-PEG-azide	200	0.063	0.037
500 nm PS	570	25	0.11
500 nm PS-PEG-azide	620	12	0.11
1 μm PS	1200	140	0.10
1 μm PS-PEG-azide	1100	52	0.061
500 nm PS-PEG-polyA20	620	52	0.097
500 nm PS-PEG-polyA40	540	6.0	0.024
500 nm PS-PEG-10% polyA20	600	13	0.20
500 nm PS-PEG-75% polyA20	620	9.1	0.12
500 nm PS-PEG-polyA20/T20	640	5.6	0.13
1 μm PS-PEG-polyA20/T20	1100	38	0.063
100 nm PS-PEG-biotin	140	0.56	0.011
200 nm PS-PEG-biotin	210	0.093	0.025
500 nm PS-PEG-10% biotin	530	17	0.037
500 nm PS-PEG-100% biotin	650	31	0.10
200 nm PS-PEG-polyA20	230	2.5	0.029

Measurements in 50 mM NaCl, 20 mM Tris, pH 7.5

Particle	Size (nm)	SD (nm)	PDI
500 nm PS-PEG-azide	540	21	0.10
500 nm PS-PEG-polyA20	620	59	0.17

Measurements in 150 mM NaCl, 20 mM Tris, pH 7.5

Particle	Size (nm)	SD (nm)	PDI
500 nm PS-PEG-azide	570	28	0.064
500 nm PS-PEG-polyA20	640	110	0.093

Measurements in 900 mM NaCl, 20 mM Tris, pH 7.5

Particle	Size (nm)	SD (nm)	PDI
500 nm PS-PEG-azide	570	6.6	0.11
500 nm PS-PEG-polyA20	580	15	0.13

Table 2. Size measurements of beads used in this study. Particle size was measured by dynamic light scattering. Particle size stated in the leftmost column is the nominal size reported by the manufacturer. Measured size and standard deviation (SD) of size are based on n = 2 independent measurements, as is the polydispersity index (PDI). For reference, PDI < 0.1 is indicative of a monodisperse sample, PDI 0.1 - 0.4 is indicative of a moderately polydisperse sample, and PDI > 0.4 is indicative of a broad polydisperse sample.

Measurements in 0.1x PBS

Particle	Charge (mV)	SD (mV)
200 nm PS	-51	5.3
200 nm PS-PEG-azide	0.11	0.40
500 nm PS	-66	1.7
500 nm PS-PEG-azide	-0.51	0.66
500 nm PS-PEG-10% polyA20	-4.7	5.8
500 nm PS-PEG-75% polyA20	-29	1.7
500 nm PS-PEG-100% polyA20	-34	4.7
500 nm PS-PEG-polyA5	-16	5.3
500 nm PS-PEG-polyA40	-37	5.8
500 nm PS-PEG-polyA20/T20	-28	0.97

Measurements in 50 mM NaCl, 20 mM Tris, pH 7.5

Particle	Charge (mV)	SD (mV)
500 nm PS-PEG-azide	-0.97	1.6
500 nm PS-PEG-100% polyA20	-16	4.6

Measurements in 150 mM NaCl, 20 mM Tris, pH 7.5

Particle	Charge (mV)	SD (mV)
500 nm PS-PEG-azide	-1.8	0.14
500 nm PS-PEG-100% polyA20	-13	0.41

Measurements in 900 mM NaCl, 20 mM Tris, pH 7.5

Particle	Charge (mV)	SD (mV)
500 nm PS-PEG-azide	3.6	0.64
500 nm PS-PEG-100% polyA20	-12	3.9

Table 3. Zeta potential measurements of beads used in this study. Zeta potential was measured by laser doppler electrophoresis. Numbers represent average of n = 2 independent measurements. SD is the standard deviation. Zeta potential of 500 nm PS-PEG-azide and 500 nm PS-PEG-100%polyA20 beads were measured in various buffer conditions used in this study.

Size ratio σ_2/σ_1	Box dimensions (units of σ)	Number of proteins	Number of beads	Total number of particles
1.0	40,40,400	51200	10240	61440
1.5	40,40,400	51200	3034	54234
2.0	40,40,400	51200	1280	52480
2.5	40,40,400	51200	655	51855
3.0	40,40,400	51200	379	51579
4.0	40,40,400	51200	160	51360

Table 4. Size and composition of MD simulations. For each size ratio of bead to protein, the simulation was conducted in the same size simulation box and kept at a constant mass ratio of bead to protein of 5:1. Thus, the number of beads decreases for larger bead sizes to keep the ratio constant.

	DNA sequence
Cy5-polyA20	5′ Cy5-A20 3′
Cy5-polyT20	5′ Cy5-T20 3′
polyA5-DBCO	5′ DBCO-A5 3′
polyA20-DBCO	5′ DBCO-A20 3′
polyA40-DBCO	5′ DBCO-A40 3′
polyT20-DBCO	5′ DBCO-T20 3′

Table 5. Complete list of the synthesized oligonucleotides.

Bead	PEG polymer	Polymer Catalog Number
PS-PEG-biotin	biotin-PEG-amine	PJK-1942
PS-PEG-oligonucleotide	azide-PEG-amine	PHB-1884
PS-PEG	mPEG-amine	PLS-268

Table 6. PEG polymers used in this paper. PEG polymers were purchased from Creative PEGWorks. PEG molecular weight was 5 kDa.

	Red Fluorescent				Yellow-Green Fluorescent			
Size	100 nm	200 nm	500 nm	1 μm	100 nm	200 nm	500 nm	1 μm
Catalog number	F8887				F8888	F8888	F8813	F8888
Lot number	2714423				2530763	2530763	2147805	2530763
Volume of beads	50 μL	50 μL	50 μL	50 μL	50 μL	50 μL	50 μL	50 μL
Mass of PEG (mg)	6.18	5.34	0.31	0.62	3.17	5.25	2.69	1.98
Mass of NHS (mg)	2.68	2.32	0.13	0.27	1.37	2.28	1.17	0.86
Vol. borate buffer (μL)	800	800	400	400	800	800	800	800
Mass of EDC (mg)	11.5	14.38	7.19	7.19	5.75	14.38	14.38	28.76
Centrifugation speed (x g)	21,000	18,000	15,000	10,000	21,000	18,000	15,000	10,000
Centrifugation time	25 min	15 min	12 min	10 min	25 min	15 min	12 min	10 min

Table 7. PEGylation reaction conditions for Fluospheres (ThermoFisher) carboxylate-modified polystyrene microspheres.

	Green Fluor.	Far Red Fluor.
Size	500 nm	500 nm
Product number	FCDG005	FCFR005
Volume of beads	50 μL	50 μL
Mass of PEG (mg)	2.44	2.44
Mass of NHS (mg)	1.06	1.06
Volume of borate buffer (μL)	800	800
Mass of EDC (mg)	14.38	14.38
Centrifugation speed (x g)	15,000	15,000
Centrifugation time	12 min	12 min

Table 8. PEGylation reaction conditions for Fluorescent Carboxyl Polystyrene beads from Bangs Labs.

Supplementary Notes

Sequences of proteins used in this paper

Throughout this paper, RGG denotes residues 1-168 of LAF-1. N protein denotes SARS-CoV-2 nucleocapsid protein. $(GRGNSPYS)_{25}$ is an artificial intrinsically disordered protein containing 25 repeats of GRGNSPYS; for brevity, $(GRGNSPYS)_{25}$ is sometimes referred to as GRGNSPYS in the paper. SA denotes streptavidin and MBP denotes maltose-binding protein. All proteins include a hexahistidine tag at the N or C-terminus for immobilized metal affinity chromatography. Below, the main domains are color-coded; tags, linkers, and cut sites are not colored.

RGG
MESNQSNNGGSGNAALNRGGRYVPPHLRGGDGGAAAAASAGGDDRRGGAGGGGYRRGGG
NSGGGGGGGYDRGYNDNRDDRDNRGGSGGYGRDRNYEDRGYNGGGGGGGNRGYNNNRG
GGGGGYNRQDRGDGGSSNFSRGGYNNRDEGSDNRGSGRSYNNDRRDNGGDGLEHHHHHH

N protein
MHHHHHHENLYFQGMSDNGPQNQRNAPRITFGGPSDSTGSNQNGERSGARSKQRRPQGLPN
NTASWFTALTQHGKEDLKFPRGQGVPINTNSSPDDQIGYYRRATRRIRGGDGKMKDLSPRWYF
YYLGTGPEAGLPYGANKDGIIWVATEGALNTPKDHIGTRNPANNAAIVLQLPQGTTLPKGFYAEG
SRGGSQASSRSSSRSRNSSRNSTPGSSRGTSPARMAGNGGDAALALLLLDRLNQLESKMSGK
GQQQQGQTVTKKSAAEASKKPRQKRTATKAYNVTQAFGRRGPEQTQGNFGDQELIRQGTDYK
HWPQIAQFAPSASAFFGMSRIGMEVTPSGTWLTYTGAIKLDDKDPNFKDQVILLNKHIDAYKTFP
PTEPKKDKKKKADETQALPQRQKKQQTVTLLPAADLDDFSKQLQQSMSSADSTQA

$(GRGNSPYS)_{25}$
MRGSHHHHHHGSRSENLYFQGRSEFDPGRGNSPYSGRGNSPYSGRGNSPYSGRGNSPYSGR
GNSPYSGRGNSPYSGRGNSPYSGRGNSPYSGRGNSPYSGRGNSPYSGRGNSPYSGRGNSPY
SGRGNSPYSGRGNSPYSGRGNSPYSGRGNSPYSGRGNSPYSGRGNSPYSGRGNSPYSGRG
NSPYSGRGNSPYSGRGNSPYSGRGNSPYSGRGNSPYSGRGNSPYSGT

MBP-SA-RGG
MHHHHHHGGGSMKIEEGKLVIWINGDKGYNGLAEVGKKFEKDTGIKVTVEHPDKLEEKFPQVAA
TGDGPDIIFWAHDRFGGYAQSGLLAEITPDKAFQDKLYPFTWDAVRYNGKLIAYPIAVEALSLIYN
KDLLPNPPKTWEEIPALDKELKAKGKSALMFNLQEPYFTWPLIAADGGYAFKYENGKYDIKDVGV
DNAGAKAGLTFLVDLIKNKHMNADTDYSIAEAAFNKGETAMTINGPWAWSNIDTSKVNYGVTVLP
TFKGQPSKPFVGVLSAGINAASPNKELAKEFLENYLLTDEGLEAVNKDKPLGAVALKSYEEELVK
DPRIAATMENAQKGEIMPNIPQMSAFWYAVRTAVINAASGRQTVDEALKDAQTNSSSNNNNNN
NNNLGENLYFQGMAEAGITGTWYNQLGSTFIVTAGADGALTGTYESAVGNAESRYVLTGRYDSA
PATDGSGTALGWTVAWKNNYRNAHSATTWSGQYVGGAEARINTQWLLTSGTTEANAWKSTLV
GHDTFTKVKPSAASLVPRGSMESNQSNNGGSGNAALNRGGRYVPPHLRGGDGGAAAAASAG
GDDRRGGAGGGGYRRGGGNSGGGGGGGYDRGYNDNRDDRDNRGGSGGYGRDRNYEDRG
YNGGGGGGGNRGYNNNRGGGGGGYNRQDRGDGGSSNFSRGGYNNRDEGSDNRGSGRSYN
NDRRDNGGDGLEHHHHHH

Derivation of LJ-based theoretical model

To determine the change in energy from insertion of a particle into a condensed phase of protein, we consider two competing effects: 1) the displacement of favorable protein-protein interactions, and 2) the addition of favorable protein-bead interactions. We ignore bead-bead interactions because we are considering the case of insertion of a bead into a dense phase purely comprising protein. This assumption is likely one reason the theoretical boundary does not line up with the crossover point calculated from simulations.

To determine the energetic component of the transfer free energy, we define:

S1. $\Delta U_{transfer} = \Delta U_{displace\ prot.} + \Delta U_{prot-bead}$

The displacement energy is assumed to be equal to the volume displaced by the bead, multiplied by the mean field internal energy of the pure protein liquid.

S2. $\Delta U_{displace\ prot.} = V_2 U_{(1)} \rho_{(1)}$

where $U_{(1)}$ is the per-particle energy of the system, and $\rho_{(1)}$ is the particle density. Note that these values are dependent on the conditions of the simulation, such as temperature. To obtain estimates for $U_{(1)}$ and $\rho_{(1)}$, we consider the pure dense phase of protein using the density profile from one case where protein-bead interactions are weak ($\varepsilon_{12} = 0.4$) and the concentration of beads inside the condensate is zero. This gives us a pure protein phase density of $\rho_{(1)} = 0.73$. The energy density of the dense phase of protein can be obtained by either running NVT simulation of pure protein in a box at density of 0.73, or can be interpolated from data already available through the NIST database [3].

The protein-bead interaction energy is assumed to be equal to a scaled-up version of a protein particle. Essentially, we take the per-particle energy from the pure protein case ($U_{(1)}$) and scale it up based on the increase in surface area (A_2/A_1), and the pairwise interaction energy ($\varepsilon_{12}/\varepsilon_1$). This gives:

S3. $\Delta U_{prot-bead} = U_{(1)} \left(\frac{A_2}{A_1}\right)\left(\frac{\epsilon_{12}}{\epsilon_1}\right)$

These are then combined to form equation 1 in the main text.

Limitations of theory applied to simulations

For small bead sizes, the partition coefficient is generally smaller than theory predicts. This may be due to several factors. First, the bead concentration at which the simulations were conducted is significantly higher than the theory accounts for, and thus the condensate environment is not purely protein. This effect would be particularly pronounced for cases with smaller beads, since the number of particles is greater than in cases with larger particles. Second, the theory assumes continuous energy density throughout the simulation box and that the attractive interactions between the bead and the protein particles will increase proportionally with respect to the surface area of the particle, which would be most valid at large bead sizes, but breaks down at small particle sizes. It also assumes that increased protein-bead interaction strength will not perturb the arrangement of protein molecules around the bead, which is also not true, but nontrivial to quantify. A more rigorous formulation may take into account the radial distribution function, which would need to be calculated separately for each case. Finally, in all cases, the concentration of protein particles in the low-density phase is significant, and will participate in attractive interactions with the beads, thus lowering the relative favorability of incorporating into the dense phase. This effect may be counteracted by reducing the temperature of the simulation, or by representing proteins as polymers. It may also be accounted for in the theoretical considerations by including a nonzero term for interactions in the dilute phase.

Supplementary References

(1) Dignon, G. L.; Zheng, W.; Kim, Y. C.; Best, R. B.; Mittal, J. Sequence Determinants of Protein Phase Behavior from a Coarse-Grained Model. *PLoS Comput. Biol.* **2018**, *14* (1), 1–23. https://doi.org/10.1371/journal.pcbi.1005941.

(2) Dignon, G. L.; Zheng, W.; Kim, Y. C.; Mittal, J. Temperature-Controlled Liquid–Liquid Phase Separation of Disordered Proteins. *ACS Cent. Sci.* **2019**, *5* (5), 821–830. https://doi.org/10.1021/acscentsci.9b00102.

(3) Daniel Siderius. NIST Standard Reference Simulation Website - SRD 173, 2017. https://doi.org/10.18434/MDS2-232.

Uncropped gel images

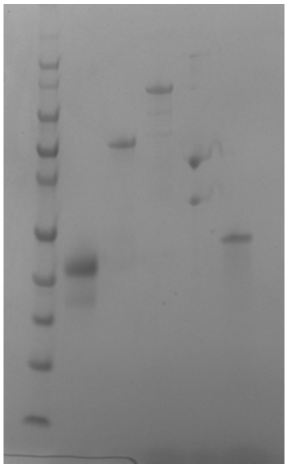

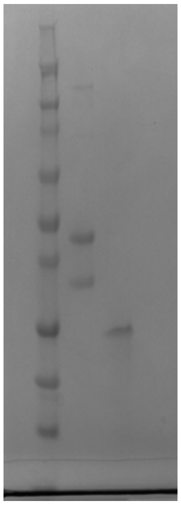

Supplement: Supplementary file 1 — Supplementary Information [file 41467_2025_58900_MOESM1_ESM.pdf]
